# Impact of Radiotherapy on Kidney Function among Patients Who Received Adjuvant Treatment for Gastric Cancer: Logistic and Linear Regression Analyses

**DOI:** 10.3390/cancers13010059

**Published:** 2020-12-28

**Authors:** Jun Su Park, Jeong Il Yu, Do Hoon Lim, Heerim Nam, Young Il Kim, Jeeyun Lee, Won Ki Kang, Se Hoon Park, Seung Tae Kim, Jung Yong Hong, Tae Sung Sohn, Jun Ho Lee, Ji Yeong An, Min Gew Choi, Jae Moon Bae

**Affiliations:** 1 Department of Radiation Oncology, Chungnam National University Sejong Hospital, Chungnam National University School of Medicine, Sejong 30099, Korea; jsrtpark@cnuh.co.kr (J.S.P.); minesota@cnuh.co.kr (Y.I.K.); 2 Department of Radiation Oncology, Samsung Medical Center, Sungkyunkwan University School of Medicine, Seoul 06351, Korea; dh8.lim@samsung.com; 3 Department of Radiation Oncology, Kangbuk Samsung Hospital, Sungkyunkwan University School of Medicine, Seoul 03181, Korea; heerim.nam@samsung.com; 4 Department of Medicine (Division of Hematology-Oncology), Samsung Medical Center, Sungkyunkwan University School of Medicine, Seoul 06351, Korea; jyun.lee@samsung.com (J.L.); wonki.kang@samsung.com (W.K.K.); sh1767.park@samsung.com (S.H.P.); seungtae1.kim@samsung.com (S.T.K.); jungyong.hong@samsung.com (J.Y.H.); 5 Department of Surgery, Samsung Medical Center, Sungkyunkwan University School of Medicine, Seoul 06351, Korea; ts.sohn@samsung.com (T.S.S.); junho3371.lee@samsung.com (J.H.L.); jar319.an@samsung.com (J.Y.A.); mingew.choi@samsung.com (M.G.C.); jmoon.bae@samsung.com (J.M.B.)

**Keywords:** stomach neoplasm, radiotherapy, chemotherapy, renal insufficiency, glomerular filtration rate

## Abstract

**Simple Summary:**

Purpose of the present study was to investigate the incidence of renal function impairment after adjuvant treatment for gastric cancer and the impact of radiotherapy on estimated glomerular filtration rate (eGFR) five years after gastric surgery. Of the 663 patients who were followed up for ≥5 years without disease recurrence and whose baseline kidney function was normal, only 2.0% of patients developed renal function impairment after adjuvant treatment for gastric cancer. While radiotherapy was negatively associated with the five-year eGFR in linear regression analysis, its impact was minimal if the kidneys were properly shielded. This study could serve as a partial basis for further research on radiation-related renal function impairment in patients who received radiotherapy for abdominal malignancy.

**Abstract:**

We investigated the incidence of renal function impairment after adjuvant treatment for gastric cancer and analyzed the impact of radiotherapy on estimated glomerular filtration rate (eGFR) five years after gastric surgery. We reviewed the medical records of 1490 patients with stomach cancer who underwent curative surgery and adjuvant treatment for gastric cancer. Finally, we included 663 patients who were followed up for ≥5 years without disease recurrence and whose baseline eGFR was ≥60 mL/min/1.73 m^2^. Logistic and linear regression analyses were performed to determine independent factors associated with the five-year eGFR. A total of 13 (2.0%) patients developed renal function impairment (five-year eGFR <60 mL/min/1.73 m^2^). In logistic regression analysis, the baseline eGFR was identified as a prognostic factor for renal function impairment (odds ratio (OR), 0.878; 95% confidence interval (CI), 0.831–0.927; *p* < 0.001), but radiotherapy was not (OR, 1.130; 95% CI, 0.366–3.491; *p* = 0.832). In linear regression analysis, age (B = −0.350, *p* < 0.001), baseline eGFR (B = 0.576, *p* < 0.001), cisplatin (B = −2.056, *p* = 0.010), and radiotherapy (B = −2.628, *p* < 0.001) were predictive variables for the five-year eGFR. Among patients who received adjuvant radiotherapy, age (B = −0.277, *p* < 0.001), hypertension (B = −4.986, *p* = 0.004), baseline eGFR (B = 0.665, *p* < 0.001), and volume of the kidneys receiving ≥20 Gy (B = −0.209, *p* = 0.012) were predictive variables for the five-year eGFR. Development of renal function impairment after adjuvant treatment for gastric cancer was rare among patients with normal baseline kidney function. While radiotherapy was negatively associated with the five-year eGFR, its impact would have been minimal if the kidneys were properly shielded. Further studies are needed to confirm the impact of radiotherapy in patients with poor kidney function.

## 1. Introduction

Stomach cancer is the most common cancer and the fourth leading cause of cancer-related deaths in South Korea [1]. Age-standardized mortality steadily decreased from 23.8 per 100,000 individuals in 1999 to 7.7 per 100,000 individuals in 2017. After curative surgery, adjuvant treatment is determined based on the pathological stage, margin status, and extent of lymph node (LN) dissection [2]. For patients with stage II or III disease, adjuvant chemotherapy is recommended after curative R0 resection with D2 LN dissection. Adjuvant chemoradiation therapy is a valid option for stomach cancer, especially in patients with R1 resection or less D2 LN dissection.

In the Adjuvant Chemoradiation Therapy in Stomach Cancer (ARTIST) trial, a randomized controlled trial comparing adjuvant chemotherapy and chemoradiation therapy after complete resection with D2 LN dissection, the most common non-hematological grade-three and -four toxicities in the chemoradiation therapy arm were nausea (12.3%), vomiting (3.1%), hand–foot syndrome (3.1%), stomatitis (1.8%), diarrhea (0.9%), and constipation (0.9%) [3]. The incidence of these toxicities was similar to that in the chemotherapy arm. Although no significant difference was found in this phase III trial, concerns remain regarding renal function impairment associated with inevitable radiation exposure of the kidney.

Since the radiation field encompasses para-aortic LNs between the upper margin of the origin of the celiac artery and the lower border of the left renal artery, the upper portion of the bilateral kidneys is also included in radiation field [4]. Several works in the literature investigated the impact of radiotherapy on kidney function in gastric cancer [5,6,7,8,9]. A similar conclusion was drawn that the higher radiation dose to the kidney was associated with the higher risk of renal function impairment. However, most of these had less than 50 subjects, and only one study in the literature included 87 subjects and reported the result of follow-up for a median of 4.7 years.

In the present study, we included patients who received adjuvant treatment for gastric cancer and were followed up for five years or longer, without disease recurrence. First, we investigated the incidence of renal function impairment five years after gastric surgery and its potential risk factors, including usage of radiotherapy. Then, the relationship between the kidney-function change over five years and the dose-volume histogram (DVH) of the kidney was analyzed with other risk factors in the subgroup receiving adjuvant radiotherapy.

## 2. Results

### 2.1. Patient Characteristics

**Figure 1:**
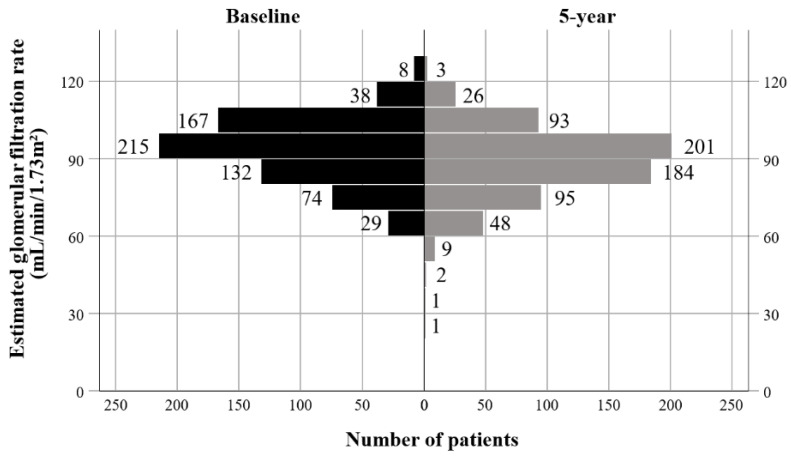
Histogram of baseline and five-year estimated glomerular filtration rates.

**Figure 2:**
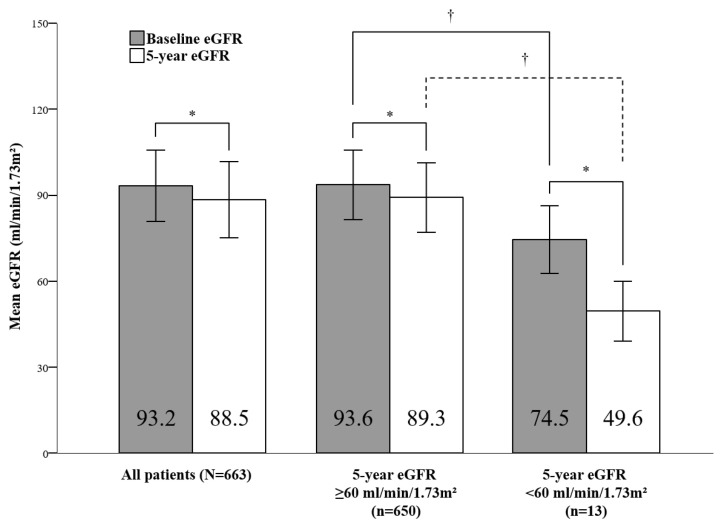
Comparison of baseline and five-year estimated glomerular filtration rates; eGFR, estimated glomerular filtration rate. * Paired *t*-test, *p* < 0.001; ^†^ independent-sample *t*-test, *p* < 0.001.

A total of 663 patients were included, and their characteristics are presented in Table 1. The median age was 53 years (range, 26–77 years). The prevalence of hypertension and diabetes was 13.6% (*n* = 90) and 10.3% (*n* = 68), respectively. In total, 183, 184, and 296 patients were treated according to the ARTIST, Adjuvant Chemotherapy Trial of TS-1 for Gastric Cancer (ACTS-GC), and Intergroup-0116 (INT-0116) trial protocols, respectively. The numbers of patients with stage IB, II, and III cancer were 72 (10.9%), 293 (44.2%), and 298 (44.9%), respectively. Most patients had LN metastasis (*n* = 639, 96.4%). A total of 389 (58.7%) patients received radiotherapy. Patients who received radiotherapy were younger (mean age 51.4 vs. 55.7 years, *p* < 0.001) and had a lower prevalence of hypertension (11.1% vs. 17.2%, *p* = 0.024) and diabetes (7.5% vs. 14.2%, *p* = 0.005), as compared to those who did not receive radiotherapy (Table 2). The mean estimated glomerular filtration rate (eGFR) before surgery and five years after surgery were 93.2 ± 12.4 mL/min/1.73 m^2^ and 88.5 ± 13.3 mL/min/1.73 m^2^, respectively (*p* < 0.001; Figure 1 and Figure 2).

### 2.2. Changes in Kidney Function and Its Associated Factors in All Patients (n = 663)

#### 2.2.1. Changes in Kidney Function and Incidence of Renal Function Impairment

A total of 13 (2.0%) patients developed renal function impairment, which was defined as an eGFR <60 mL/min/1.73 m^2^. The number of patients who received radiotherapy was eight, and the detailed characteristics are presented in Table S1. The lowest five-year eGFR was 25.7 mL/min/1.73 m^2^, and the largest decrease in eGFR was from 91.2 mL/min/1.73 m^2^ to 30.6 mL/min/1.73 m^2^. The mean age of these 13 patients was higher than that of the other 650 patients whose eGFR remained normal (62.8 ± 8.6 years vs. 53.0 ± 10.6 years, *p* = 0.001). The prevalence of hypertension and diabetes was higher in these 13 patients than in the other 650 patients (46.2% vs. 12.9%, *p* = 0.004 and 30.8% vs. 9.8%, *p* = 0.036, respectively). The mean eGFR decreased from 74.5 ± 11.8 mL/min/1.73 m^2^ to 49.6 ± 10.5 mL/min/1.73 m^2^ (*p* < 0.001) among patients who developed renal function impairment, while it decreased from 93.6 ± 12.2 mL/min/1.73 m^2^ to 89.3 ± 12.1 mL/min/1.73 m^2^ (*p* < 0.001) among patients whose eGFR remained normal (Figure 2). There were no statistically significant differences in the use of cisplatin or radiotherapy as adjuvant treatments (*p* = 0.205 and *p* = 0.832, respectively).

#### 2.2.2. Logistic Regression Analysis to Predict a Development of Renal Function Impairment

In the univariate analysis, continuous variables, including age (odds ratio (OR), 1.109; 95% confidence interval (CI), 1.038–1.184; *p* = 0.002) and baseline eGFR (OR, 0.878; 95% CI, 0.831–0.927; *p* < 0.001), were associated with the development of renal function impairment. Categorical variables, including hypertension (OR, 5.776; 95% CI, 1.895–17.600; *p* = 0.002) and diabetes (OR, 4.069; 95% CI, 1.219–13.589; *p* = 0.023), were also relevant. However, sex (OR, 1.040; 95% CI, 0.336–3.215; *p* = 0.946), cisplatin (OR, 2.291; 95% CI, 0.759–6.909; *p* = 0.141), and radiotherapy (OR, 1.130; 95% CI, 0.366–3.491; *p* = 0.832) were not relevant. In the multivariate analysis analyzing all variables that attained statistical significance in the univariate analysis, only baseline eGFR remained a significant prognostic factor of renal function impairment (OR, 0.878; 95% CI, 0.831–0.927; *p* < 0.001).

#### 2.2.3. Linear Regression Analysis to Predict Five-Year eGFR

The results of the multiple linear regression analysis are presented in Table 3. Radiotherapy along with age, cisplatin, and baseline eGFR were associated with the five-year eGFR (adjusted R^2^ = 0.528, *p* < 0.001). The younger the age or the higher the baseline eGFR, the higher the five-year eGFR (B = −0.350, *p* < 0.001 and B = 0.576, *p* < 0.001, respectively). Radiotherapy and cisplatin were negative predictive variables for the five-year eGFR (B = −2.628, *p* < 0.001 and B = −2.056, *p* = 0.010, respectively).

#### 2.2.4. Logistic Regression Analysis to Predict a Decrease of 10% or More in eGFR

A logistic regression analysis was performed to predict a decrease of ≥10% in eGFR from the baseline value, which occurred in 25.9% of total patients (*n* = 172). In multivariate analysis, age (OR, 1.048; 95% CI, 1.025–1.071; *p* < 0.001), radiotherapy (OR, 1.557; 95% CI, 1.069–2.268; *p* = 0.021), and baseline eGFR (OR, 1.046; 95% CI, 1.026–1.066; *p* < 0.001) were associated with the decrease in five-year eGFR (Table S2). The nomogram predicting a decrease of ≥10% in eGFR from the baseline value was constructed based on these variables (Figure S1A).

### 2.3. Changes in Kidney Function and Its Associated Factors in Subgroup (n = 287)

Among 389 patients who received radiotherapy, DVH data were available in 287 patients. A subgroup analysis exploring the quantitative correlation between kidney volume (%) receiving ≥5 Gy and ≥20 Gy (V_5Gy_ and V_20Gy_, respectively), mean radiation dose (Gy) to the kidney (D_mean_), and five-year eGFR was performed in these patients. The median kidney V_5Gy_, V_20Gy_, and D_mean_ were 35.9% (range, 6.0–70.8%), 14.9% (range, 0.7–38.0%), and 8.2 Gy (range, 1.8–16.3 Gy), respectively (Figures S2 and S3). The logistic regression analysis to predict a development of renal function impairment was not performed, since only four patients developed renal function impairment in subgroup.

#### 2.3.1. Linear Regression Analysis to Predict Five-Year eGFR

In the multivariate analysis, age, hypertension, baseline eGFR, and kidney V_20Gy_ were associated with the five-year eGFR (adjusted R^2^ = 0.585, *p* < 0.001) (Table 3). Similar to the result of the analysis of all patients, the younger the age or the higher the baseline eGFR, the higher the five-year eGFR (B = −0.277, *p* < 0.001 and B = 0.665, *p* < 0.001, respectively). Although kidney V_5Gy_ and D_mean_ were not associated with the five-year eGFR, kidney V_20Gy_ (B = −0.209, *p* = 0.012) and hypertension (B = −4.986, *p* = 0.004) were negative predictive variables for the five-year eGFR.

#### 2.3.2. Logistic Regression Analysis to Predict a Decrease of 10% or More in eGFR

The five-year eGFR was decreased by 10% or more from baseline value in 80 patients (27.9%). In multivariate analysis, diabetes (OR, 2.723; 95% CI, 1.055–7.026; *p =* 0.038) and kidney V_20Gy_ (OR, 1.077; 95% CI, 1.031–1.124; *p* = 0.001) were associated with the decrease in five-year eGFR (Table S2). The nomogram predicting a decrease of ≥ 10% in eGFR from the baseline value was constructed based on these variables (Figure S1B).

## 3. Discussion

In the present study evaluating the incidence and risk factors of renal function impairment at five years after surgery and adjuvant treatment for gastric cancer, the incidence of new-onset renal function impairment was very low, at 2.0% (*n* = 13). Baseline eGFR was the sole prognostic factor for renal function impairment in the multivariate logistic regression analysis (OR, 0.878; 95% CI, 0.831–0.927; *p* < 0.001). Radiotherapy was not a prognostic factor in logistic regression analysis, but it was a predictive variable for the five-year eGFR in linear regression analysis (B = −2.628, *p* < 0.001). Furthermore, kidney V_20Gy_ was a negative predictive variable among patients who received adjuvant radiotherapy. However, its coefficient was quite small (B = −0.209, *p* = 0.012); when the kidney V_20Gy_ was increased by 1%, the five-year eGFR decreased by 0.209 mL/min/1.73 m^2^. We assumed that, although the impact of radiotherapy on the five-year eGFR was significant, it was too small to lead to renal function impairment.

To date, glomerular filtration rates (GFR) remains the best index of kidney function. It can be determined by measuring the urinary clearance of exogenous filtration markers or be estimated from serum creatinine levels. Although the former method is considered the gold standard, the latter method is preferred because of its convenience. There are three estimation equations, namely the Cockcroft–Gault equation, Modification of Diet in Renal Disease (MDRD) study equation, and Chronic Kidney Disease Epidemiology Collaboration (CKD-EPI) equation [10,11,12]. The CKD-EPI equation performed better than the MDRD study equation among individuals with a normal GFR and had similar accuracy among those with low GFR. Hence, we used the CKD-EPI equation to estimate the GFR. However, doubts regarding the accuracy of eGFR using serum creatinine levels remained. During the initial decrease in GFR, tubular secretion of creatinine enhances, which can alleviate the increase in serum creatinine levels. Until the tubular secretory capacity is saturated, serum creatinine levels can remain normal, and eGFR could be overestimated [13,14].

Kidneys are dose-limiting organs in radiotherapy, and the estimated radiation dose with 5% risk of nephrosclerosis at five years (TD 5/5) is 23 Gy for the whole kidney [15]. The tolerance dose was much lower at 9.8 Gy in patients receiving total body irradiation [16]. The Quantitative Analyses of Normal Tissue Effects in the Clinic study suggested dose-volume constraints for an estimated risk of <5% as follows: D_mean_ < 18 Gy and V_20Gy_ < 32% when the partial kidney is irradiated [17]. In the present study, the kidneys were shielded as much as possible, without compromising the target volume coverage, and the median kidney D_mean_ and V_20Gy_ were 8.2 Gy and 14.9%, respectively, which were much lower than the constraints.

Along with radiotherapy, cisplatin was a negative predictive variable for the five-year eGFR (B = −2.056, *p* = 0.010). Cisplatin is excreted by the kidneys, but it can also be transported into renal epithelial cells, leading to kidney injury [18]. Typical cisplatin-induced nephrotoxicity occurs within several days after administration and gradually recovers over two to four weeks. In a study on the head and neck cancer, serum creatinine levels were increased by ≥1.5 times or ≥0.3 mg/dL after receiving concurrent chemoradiation therapy with high-dose cisplatin (100 mg/m^2^) in 69% of patients, even though radiotherapy was delivered to the head and neck [19]. In contrast, 60 mg/m^2^ cisplatin was administered before and after radiotherapy in the present study. Although the dosage of cisplatin was much lower than that in the former study, the development of kidney injury from sequential administration of cisplatin and radiotherapy might have been more severe than injury from individual treatment modalities. However, cisplatin was not included as a variable in the subgroup analysis, since patients receiving cisplatin were treated with two-dimensional radiotherapy; hence, we could not obtain DVH data. If we were able to obtain DVH data of patients receiving cisplatin, we could have compared the impact of cisplatin and radiation dose on kidney function in more detail.

In addition to treatment-related factors, several patient factors affect kidney function. In general, kidney function gradually decreases with age. The Baltimore Longitudinal Study of Aging showed a mean decrease in creatinine clearance of 0.75 mL/min/year among 254 healthy subjects [20]. The annual decrease in creatinine clearance (mL/min/year) was proportional to age: −0.32 in subjects in their 40s, −0.57 in subjects in their 50s, −1.24 in subjects in their 60s, −1.49 in subjects in their 70s, and −3.25 in subjects in their 80s. Additionally, the mean blood pressure correlated with the rate of decline in creatinine clearance (*p* < 0.001) [21]. Other studies have also shown that comorbid conditions, including hypertension and heart failure, can accelerate the decline of kidney function [22,23]. The present study also showed that hypertension had a negative association with the five-year eGFR (B = −4.986, *p* = 0.004); however, this result requires careful interpretation because hypertension and diabetes were determined based only on the history of the patient’s medication. To investigate the impact of comorbid diseases, it would have been more appropriate to investigate the duration and control status of comorbid diseases.

This study had several limitations. First, this was a retrospective study among patients with locally advanced stomach cancer. Second, although the radiotherapy scheme was identical throughout the study period, there were three different adjuvant treatment strategies, and the choice depended on the time of surgery. Patients were randomly assigned to receive chemotherapy with radiotherapy or chemotherapy alone in the ARTIST trial, while it was determined according to patient preference in those who were treated after the closure of the ARTIST trial. Hence, there were significant differences in patient characteristics between those who received radiotherapy and those who did not (Table 2). Patients who received radiotherapy were relatively younger and had a lower prevalence of hypertension and diabetes, as compared to those who did not receive radiotherapy. Third, the radiation field in the present study was different from the traditional radiation field, which included the remnant stomach after subtotal gastrectomy. We did not include the remnant stomach in the radiation field based on the findings of a previous study by Nam et al. [24], which retrospectively compared the treatment results between two different radiation fields, including and excluding the remnant stomach after subtotal gastrectomy. Exclusion of the remnant stomach did not compromise the local control rate and survival, and it had fewer complications, such as grade-three or -four vomiting and diarrhea. If the remnant stomach would have been included in the radiation field as a traditional field, the upper portion of the left kidney could have received more radiation. Finally, radiotherapy was planned on the basis of images acquired during free-breathing. However, the kidneys move during respiration, and their maximal vertical motion was reported as 39 mm in a study using magnetic resonance imaging [25]. Thus, the actual radiation dose to the kidney could be different from DVH data.

## 4. Materials and Methods

### 4.1. Patients

We reviewed the medical records of 1490 patients with stomach cancer who underwent curative surgery and adjuvant treatment at Samsung Medical Center between October 2004 and February 2013. Among them, patients who were followed up for ≥5 years without disease recurrence were included, because recurrent disease itself and/or treatment for recurrent disease can affect kidney function. Patients whose baseline serum creatinine levels were not available or whose baseline eGFR was <60 mL/min/1.73 m^2^ were excluded. Patients whose serum creatinine levels were not available 5 years after surgery were also excluded.

### 4.2. Treatment

Adjuvant treatment varied according to the time of surgery (Table S3). Patients who underwent curative surgery between October 2004 and February 2008 and consented to participate were included in the ARTIST trial [3]. Thereafter, two different treatment protocols of the ACTS-GC and the INT-0116 trial were used [26,27].

The ARTIST trial was a phase III trial comparing adjuvant XP (capecitabine and cisplatin) chemotherapy alone and XP with radiotherapy (XP/XRT/XP). The XP arm administered 6 cycles of XP chemotherapy (consisting of 2000 mg/m^2^ capecitabine per day on days 1–14 and 60 mg/m^2^ cisplatin on day 1) every 3 weeks. The XP/XRT/XP arm administered 2 cycles of XP chemotherapy, followed by 45 Gy of radiotherapy with 1650 mg/m^2^ capecitabine per day for 5 weeks, followed by 2 additional cycles of XP chemotherapy. Radiotherapy was administered by using the anterior–posterior/posterior–anterior technique. The target of radiotherapy was the tumor bed, anastomosis site, duodenal stump, regional LNs, and 2 cm beyond the proximal and distal resection margins. The remnant stomach was not routinely included within the radiation field. The radiation dose was 45 Gy, with daily fractions of 1.8 Gy delivered over 5 weeks. The DVH data were not available, since two-dimensional radiotherapy was used in this period.

After the accrual of the ARTIST trial was completed, adjuvant chemotherapy alone, according to the protocol of the ACTS-GC trial, or chemoradiation therapy, according to the INT-0116 trial, was administered based on patient preference with informed consent. In the adjuvant chemotherapy alone protocol, 40 mg/m^2^ of S-1 was administered orally, twice a day, for 4 weeks, followed by 2 weeks of rest. This 6-week cycle was repeated during the first year after surgery. In the chemoradiation therapy protocol, first-cycle chemotherapy comprising 425 mg/m^2^ fluorouracil per day and 20 mg/m^2^ leucovorin per day was administered for 5 days, without radiotherapy. Second- and third-cycle chemotherapies comprising 400 mg/m^2^ fluorouracil per day and 20 mg/m^2^ leucovorin per day were administered on the first four and the last three days of radiotherapy, respectively. One month after radiotherapy, two 5-day cycles of fluorouracil and leucovorin were administered 1 month apart. The radiation field and dose were identical to those of the ARTIST trial. However, patients who were treated in this period received three-dimensional conformal radiotherapy, and their DVH data were available.

### 4.3. Kidney Function

Kidney function was determined by eGFR, using the CKD-EPI equation [12]. Serum creatinine levels within 3 months before surgery and 5 years after surgery were obtained. Development of renal function impairment was defined as a five-year eGFR <60 mL/min/1.73 m^2^. Since the number of patients who developed renal function impairment was small, a decrease of ≥10% in eGFR was additionally analyzed. The 10% came from the first quartile value of five-year eGFR divided by baseline eGFR.

### 4.4. Dose-Volume Histogram Data

To quantitatively evaluate the impact of radiotherapy on kidney function, we obtained the kidney V_5Gy_, V_20Gy_, and D_mean_.

### 4.5. Statistical Analysis

The baseline and five-year eGFRs were compared by using the paired *t*-test. The Chi-squared test was used to compare characteristics between patients whose five-year eGFR was normal or had decreased. We conducted logistic regression analysis to determine independent prognostic factors for the development of renal function impairment. In linear regression analysis, five-year eGFR was analyzed as a continuous variable, and categorical variables, including sex, hypertension, diabetes, cisplatin, and radiotherapy, were coded into binary variables (0 and 1). A *p*-value < 0.05 indicated statistical significance. All statistical analyses were performed by using SPSS version 24 (IBM, Armonk, NY, USA). Nomogram was drawn by using R version 3.6.1 (http://www.r-project.org).

## 5. Conclusions

In the present study evaluating the incidence and risk factors of renal function impairment at five years after surgery and adjuvant treatment for gastric cancer, renal function impairment (eGFR < 60 mL/min/1.73 m^2^) after adjuvant treatment for gastric cancer was rare. Radiotherapy, low baseline eGFR, old age, cisplatin, and hypertension were negatively associated with the five-year eGFR. However, if the kidneys would have been properly shielded and kidney V_20Gy_ kept low, the impact of radiotherapy on the five-year eGFR could be minimal. Thus, every effort should be made to minimize the radiation dose received by the kidneys without compromising the quality of radiotherapy. Since the present study was limited to patients with normal kidney function, further studies are needed to confirm the impact of radiotherapy in patients with poor kidney function.

## Figures and Tables

**Table 1:** Patient characteristics.

Characteristic	All (*n* = 663)	Five-Year eGFR (mL/min/1.73 m^2^)		*p*
		≥60 (*n* = 650)	<60 (*n* = 13)	
Mean Age (years)	53.2 ± 10.6	53.0 ± 10.6	62.8 ± 8.6	0.001 ^a^
Sex				
Male	414 (62.4%)	406 (62.5%)	8 (61.5%)	>0.999
Female	249 (37.6%)	244 (37.5%)	5 (38.5%)	
Hypertension				
Yes	90 (13.6%)	84 (12.9%)	6 (46.2%)	0.004 ^a^
No	573 (86.4%)	566 (87.1%)	7 (53.8%)	
Diabetes				
Yes	68 (10.3%)	64 (9.8%)	4 (30.8%)	0.036 ^a^
No	595 (89.7%)	586 (90.2%)	9 (69.2%)	
Chemotherapy				
5-fluorouracil	296 (44.6%)	292 (44.9%)	4 (30.8%)	0.312
TS-1	184 (27.8%)	181 (27.8%)	3 (23.1%)	
XP	183 (27.6%)	177 (27.2%)	6 (46.2%)	
Cisplatin				
Yes	183 (27.6%)	177 (27.2%)	6 (46.2%)	0.205
No	480 (72.4%)	473 (72.8%)	7 (53.8%)	
Radiotherapy				
Yes	389 (58.7%)	381 (58.6%)	8 (61.5%)	0.832
No	274 (41.3%)	269 (41.4%)	5 (38.5%)	
Baseline eGFR (mL/min/1.73 m^2^)	93.2 ± 12.4	93.6 ± 12.2	74.5 ± 11.8	<0.001 ^a^
Five-year eGFR (mL/min/1.73 m^2^)	88.5 ± 13.3	89.3 ± 12.1	49.6 ± 10.5	<0.001 ^a^

XP, capecitabine and cisplatin; eGFR, estimated glomerular filtration rate. ^a^ The *p*-value was <0.005.

**Table 2:** Patient characteristics according to radiotherapy.

Characteristic	All (*n* = 663)	Radiotherapy		*p*
		No (*n* = 274)	Yes (*n* = 389)	
Mean Age (years)	53.2 ± 10.6	55.7 ± 11.2	51.4 ± 9.9	0.001 ^a^
Sex				
Male	414 (62.4%)	173 (63.1%)	241 (62.0%)	0.756
Female	249 (37.6%)	101 (36.9%)	148 (38.0%)	
Hypertension				
Yes	90 (13.6%)	47 (17.2%)	43 (11.1%)	0.024 ^a^
No	573 (86.4%)	227 (82.8%)	346 (88.9%)	
Diabetes				
Yes	68 (10.3%)	39 (14.2%)	29 (7.5%)	0.005 ^a^
No	595 (89.7%)	235 (85.8%)	360 (92.5%)	
Chemotherapy				
5-fluorouracil	296 (44.6%)	9 (3.3%)	287 (73.8%)	<0.001 ^a^
TS-1	184 (27.8%)	184 (67.2%)	0 (0.0%)	
XP	183 (27.6%)	81 (29.6%)	102 (26.2%)	
Cisplatin				
Yes	183 (27.6%)	81 (29.6%)	102 (26.2%)	0.343
No	480 (72.4%)	193 (70.4%)	287 (73.8%)	
Baseline eGFR (mL/min/1.73 m^2^)	93.2 ± 12.4	92.5 ± 12.3	93.8 ± 12.5	0.176
Five-year eGFR (mL/min/1.73 m^2^)	88.5 ± 13.3	88.7 ± 12.9	88.4 ± 13.6	0.294

XP, capecitabine and cisplatin; eGFR, estimated glomerular filtration rate. ^a^ The *p*-value was <0.005.

**Table 3:** Results of multiple linear regression analysis.

Variable	Unstandardized Coefficients		Standardized Coefficients		*p*	Collinearity Statistics	
	B	SE	Beta	T		Tolerance	VIF
All patients (*n* = 663)							
Constant	53.959	4.900		11.285	<0.001		
Age (continuous)	−0.350	0.041	−0.280	16.741	<0.001	0.684	1.461
Cisplatin (0 = N, 1 = Y)	−2.056	0.798	−0.069	−8.471	0.010	0.653	1.532
Radiotherapy (0 = N, 1 = Y)	−2.628	0.737	−0.098	−3.568	<0.001	0.953	1.049
Baseline eGFR (continuous)	0.576	0.034	0.540	−2.578	<0.001	0.986	1.014
Patients who received radiotherapy and whose DVH data were available (*n* = 287)							
Constant	44.350	6.502		6.821	<0.001		
Age (continuous)	−0.277	0.060	−0.204	−4.584	<0.001	0.732	1.365
Hypertension (0 = N, 1 = Y)	−4.986	1.736	−0.112	−2.872	0.004	0.951	1.051
Kidney V_20Gy_ (continuous)	−0.209	0.083	−0.098	−2.525	0.012	0.963	1.038
Baseline eGFR (continuous)	0.665	0.047	0.630	14.133	<0.001	0.730	1.371

SE, standard error; VIF, variance inflation factor; eGFR, estimated glomerular filtration rate; DVH, dose-volume histogram; V_20Gy_, volume (%) receiving 20 Gy or higher.

## Data Availability

Data availability is limited due to institutional data protection law and confidentiality of patient data.

## Supplementary Materials

The following are available online at https://www.mdpi.com/2072-6694/13/1/59/s1. Table S1: Characteristics of 13 patients whose five-year estimated glomerular filtration rate was below 60 mL/min/1.73 m^2^. Table S2: Logistic regression analyses to predict a decrease of 10% or more in estimated glomerular filtration rate. Table S3: Comparison of the protocols of three randomized controlled trials. Figure S1: Nomograms for predicting a decrease of 10% or more in estimated glomerular filtration rate in all patients (1A) and in patients who received radiotherapy and whose DVH was available (*n* = 297). Figure S2: Histograms of the volume of kidney receiving ≥5 Gy (V_5Gy_) and ≥20 Gy (V_20Gy_). Figure S3: Histogram of mean radiation dose (D_mean_) to kidney.
