# Supplementary material for: Impact of Radiotherapy on Kidney Function among Patients Who Received Adjuvant Treatment for Gastric Cancer: Logistic and Linear Regression Analyses
Source: Cancers (Basel). 2020 Dec 28;13(1):59. doi: 10.3390/cancers13010059 (PMC7794775; doi:10.3390/cancers13010059)
Supplement: Supplementary file 1 [file cancers-13-00059-s001.pdf]

Supplementary Materials: Impact of Radiotherapy on Kidney Function among Patients Who Received Adjuvant Treatment for Gastric Cancer: Logistic and Linear Regression Analyses

Jun Su Park, Jeong Il Yu, Do Hoon Lim, Heerim Nam, Young Il Kim, Jeeyun Lee, Won Ki Kang, Se Hoon Park, Seung Tae Kim, Jung Yong Hong, Tae Sung Sohn, Jun Ho Lee, Ji Yeong An, Min Gew Choi and Jae Moon Bae

Table S1. Characteristics of 13 patients whose 5-year estimated glomerular filtration rate was below 60 mL/min/1.73 m^2.

eGFR (mL/min/1.73 m^2)								Kidney	
Baseline	5-year	Sex	Age	Hypertension	Diabetes	Chemotherapy	Radiotherapy	V_{20Gy}	D_{mean}
65.0	59.6	M	71	N	N	XP	N	NA	NA
95.7	56.7	F	59	N	N	XP	N	NA	NA
68.0	56.6	M	74	N	N	TS-1	N	NA	NA
64.4	52.8	M	77	Y	Y	TS-1	N	NA	NA
70.0	48.8	M	70	Y	Y	TS-1	N	NA	NA
68.1	59.9	M	66	Y	N	XP	Y	NA	NA
81.8	54.4	M	51	N	N	5-FU	Y	4.1%	4.9 Gy
66.7	54.3	F	62	Y	N	5-FU	Y	13.8%	8.2 Gy
68.4	50.7	M	55	N	N	XP	Y	NA	NA
93.8	50.0	F	51	N	N	XP	Y	NA	NA
63.1	44.8	F	54	Y	N	5-FU	Y	11.8%	6.5 Gy
91.2	30.6	M	64	N	Y	XP	Y	NA	NA
72.4	25.7	F	62	Y	Y	5-FU	Y	19.2%	9.8 Gy

eGFR, estimated glomerular filtration rate; XP capecitabien and cisplatin; NA, not available; 5-FU, 5-fluorouracil; V_{20Gy}, volume receiving 20 Gy or higher; D_{mean}, average radiation dose received by specific organ

Table S2. Logistic regression analyses to predict a decrease of 10% or more in estimated glomerular filtration rate.

Variable	Univariate			Multivariate		
	OR	95% CI	*p*	OR	95% CI	*p*
**All patients (*N* = 663)**						
Age (continuous)	1.012	0.996-1.029	0.141	1.048	1.025-1.071	< 0.001
Sex (female)	0.886	0.617-1.271	0.511			
Hypertension	1.345	0.829-2.184	0.230			
Diabetes	1.031	0.584-1.821	0.917			
Cisplatin	1.237	0.845-1.810	0.274			
Radiotherapy	1.348	0.942-1.930	0.103	1.557	1.069-2.268	0.021
Baseline eGFR (continuous)	1.022	1.007-1.037	0.004	1.046	1.026-1.066	<0.001
**Patients who received radiotherapy and whose DVH data was available (*n* = 287)**						
Age (continuous)	1.020	0.993-1.048	0.156			
Sex (female)	1.733	1.026-2.928	0.040			
Hypertension	1.782	0.795-3.993	0.161			
Diabetes	2.259	0.898-5.678	0.083	2.723	1.055-7.026	0.038
Baseline eGFR (continuous)	1.020	0.999-1.042	0.066			
Kidney V_{5Gy} (continuous)	1.023	0.997-1.051	0.082			
Kidney V_{20Gy} (continuous)	1.071	1.027-1.118	0.002	1.077	1.031-1.124	0.001
Kidney D_{mean} (continuous)	1.002	1.001-1.003	0.004			

OR, odds ratio; CI, confidence interval; eGFR, estimated glomerular filtration rate; DVH, dose-volume histogram; V_{5Gy}, volume receiving 5 Gy or higher; V_{20Gy}, volume receiving 20 Gy or higher; D_{mean}, average radiation dose received by specific organ

Table S3. Comparison of the protocols of three randomized controlled trials.

Trial	Intervention	Chemotherapy regimen	Radiation therapy	Radiation dose
ARTIST	Adjuvant CTx *vs.* CCRT	Capecitabine and cisplatin	Y/N (1:1)	45 Gy/25 fractions
ACTS-GC	Adjuvant CTx *vs.* observation	TS-1	N	
INT-0116	Adjuvant CCRT *vs.* observation	5-fluorouracil	Y	45 Gy/25 fractions

ARTIST, Adjuvant Chemoradiation Therapy in Stomach Cancer; CTx, chemotherapy; CCRT, concurrent chemoradiation therapy; ACTS-GC, Adjuvant Chemotherapy Trial of TS-1 for Gastric Cancer; INT, Intergroup

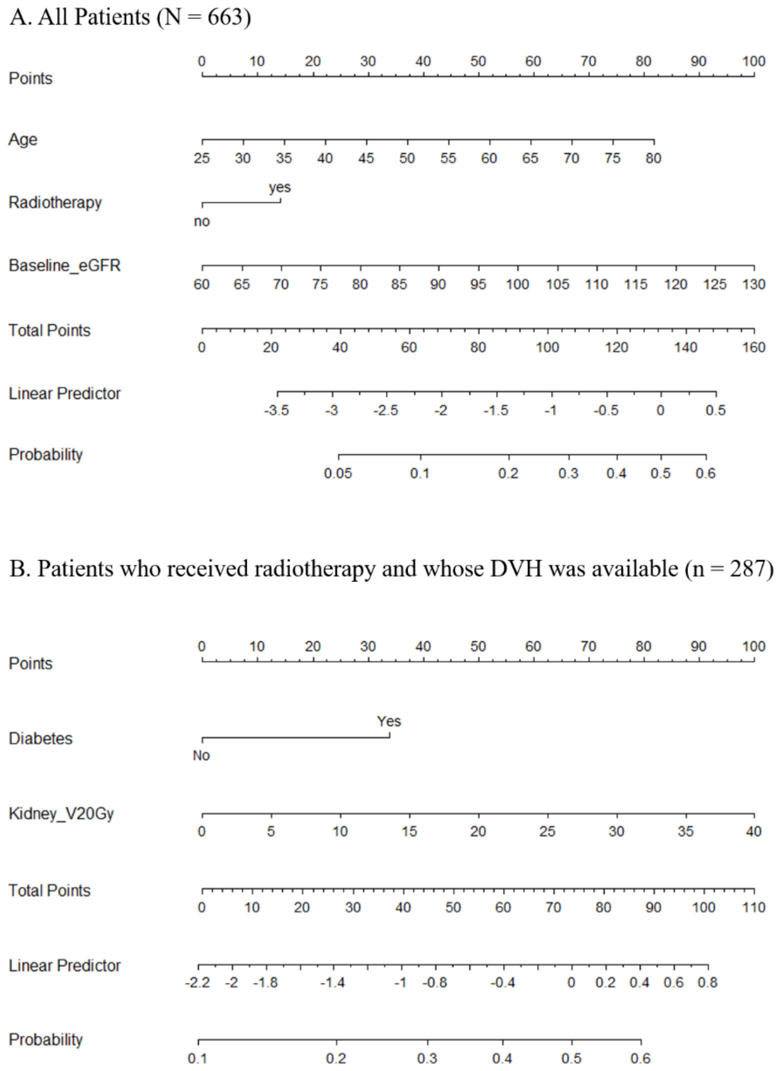

Figure S1. Nomograms for predicting a decrease of 10% or more in estimated glomerular filtration rate in all patients (1A) and in patients who received radiotherapy and whose DVH was available (n = 297).

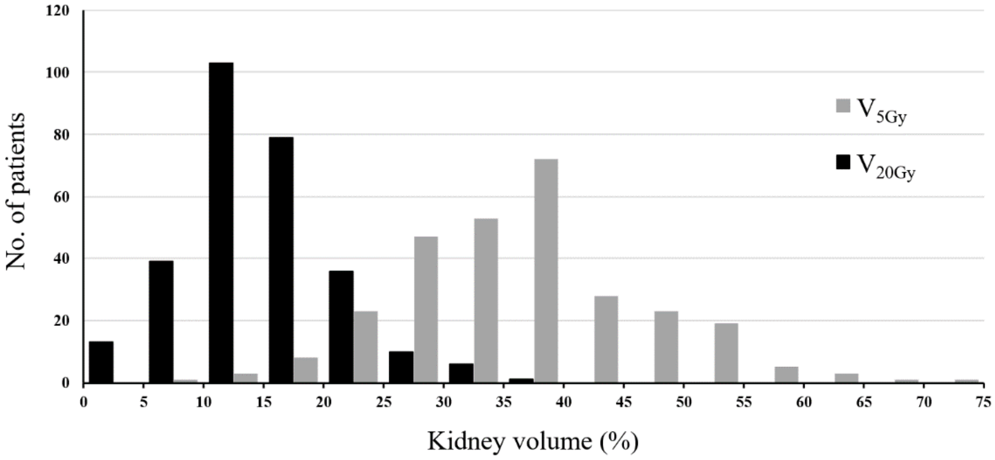

Figure S2. Histograms of the volume of kidney receiving ≥5 Gy (V_{5Gy}) and ≥20 Gy (V_{20Gy}).

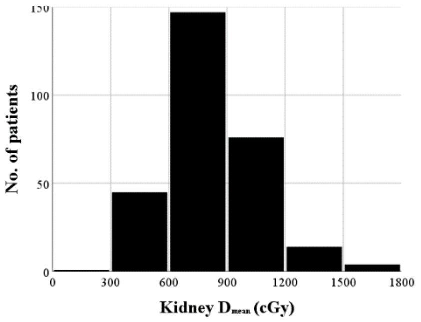

Figure S3. Histogram of mean radiation dose (D_{mean}) to kidney.

Copyright: © 2020 by the author. Licensee MDPI, Basel, Switzerland. This article is an open access article distributed under the terms and conditions of the Creative Commons Attribution (CC BY) license (http://creativecommons.org/licenses/by/4.0/).
